# Adenylate Kinase and AMP Signaling Networks: Metabolic Monitoring, Signal Communication and Body Energy Sensing

**DOI:** 10.3390/ijms10041729

**Published:** 2009-04-17

**Authors:** Petras Dzeja, Andre Terzic

**Affiliations:** Division of Cardiovascular Disease, Department of Medicine, Mayo Clinic, Rochester, MN 55905, USA; E-Mail: terzic.andre@mayo.edu (A.T.)

**Keywords:** Phosphotransfer enzymes, system bioenergetics, energy transfer, adenosine 5’-triphosphate, adenosine 5’-monophosphate, nucleotide metabolism, AMP-activated protein kinase, ATP-sensitive potassium channel

## Abstract

Adenylate kinase and downstream AMP signaling is an integrated metabolic monitoring system which reads the cellular energy state in order to tune and report signals to metabolic sensors. A network of adenylate kinase isoforms (AK1-AK7) are distributed throughout intracellular compartments, interstitial space and body fluids to regulate energetic and metabolic signaling circuits, securing efficient cell energy economy, signal communication and stress response. The dynamics of adenylate kinase-catalyzed phosphotransfer regulates multiple intracellular and extracellular energy-dependent and nucleotide signaling processes, including excitation-contraction coupling, hormone secretion, cell and ciliary motility, nuclear transport, energetics of cell cycle, DNA synthesis and repair, and developmental programming. Metabolomic analyses indicate that cellular, interstitial and blood AMP levels are potential metabolic signals associated with vital functions including body energy sensing, sleep, hibernation and food intake. Either low or excess AMP signaling has been linked to human disease such as diabetes, obesity and hypertrophic cardiomyopathy. Recent studies indicate that derangements in adenylate kinase-mediated energetic signaling due to mutations in AK1, AK2 or AK7 isoforms are associated with hemolytic anemia, reticular dysgenesis and ciliary dyskinesia. Moreover, hormonal, food and antidiabetic drug actions are frequently coupled to alterations of cellular AMP levels and associated signaling. Thus, by monitoring energy state and generating and distributing AMP metabolic signals adenylate kinase represents a unique hub within the cellular homeostatic network.

## Contents

1. Introduction
2. Adenylate kinase isoform-based energetic and metabolic signaling network
  2.1. Adenylate kinase isoforms in the nucleus
  2.2. Adenylate kinase isoforms in cell motility and nucleotide pool homeostasis
  2.3. Adenylate kinase localization and interacting partners
3. Adenylate kinase catalyzed β-phosphoryl transfer and cell energy economy
4. Adenylate kinase and AMP signaling: an integrated metabolic monitoring and signal ommunication system
5. AMP as universal fuel consumption and low energy signal, mediator of drug action and therapeutic agent
6. Adenylate kinase and AMP signaling networks in body energy sensing
7. Adenylate kinase never rests: from altered energetic signaling to immunodeficiency, cell motility defects, reticular dysgenesis and sensorineural deafness
8. Summary

## 1. Introduction

**Figure 1.**
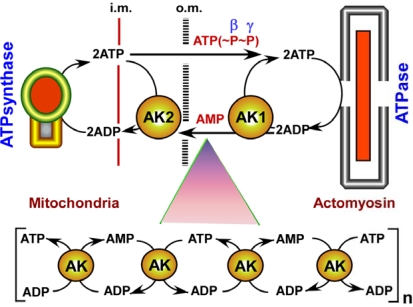
Adenylate kinase shuttle facilitates transfer of ATP β- and γ-phosphoryls from generation to utilization sites. Adenylate kinase (AK), present in mitochondrial and myofibrillar compartments, enables the transfer and makes available the energy of two high-energy phosphoryls, the β- and the γ-phosphoryls of a single ATP molecule. In this case, AMP signals feedback to mitochondrial respiration amplified by the generation of two molecules of ADP at the mitochondrial intermembrane site. Within the intracellular environment of a cardiomyocyte, the transfer of ATP and AMP between ATP-production and ATP-consumption sites may involve multiple, sequential, phosphotransfer relays that result in a flux wave propagation along clusters of adenylate kinase molecules (lower panel). Handling of substrates by “bucket-brigade” or a ligand conduction mechanism facilitates metabolic flux without apparent changes in metabolite concentrations. AK1 and AK2 – cytosolic and mitochondrial AK isoforms, respectively. i.m. and o.m. – inner and outer membranes, respectively. Modified from [5] with permission.

Recent studies provide new evidence of the unique energetic, metabolic monitoring and signaling role played by the ubiquitous enzyme adenylate kinase which catalyzes the nucleotide phosphoryl exchange reaction 2ADP ↔ ATP + AMP, critical in cell life [1–11]. Historically, the function of adenylate kinase has been ascribed to *de novo* adenine nucleotide synthesis and cell energy economy through regulation of nucleotide ratios in different intracellular compartments and AMP-sensitive metabolic enzymes [1,12–17]. Adenylate kinase has been intensely studied, including its genetics and gene polymorphism, tissue and developmental expression, intracellular distribution and structurefunction relationship [2,3,14,16–25]. This exemplifies adenylate kinase as a model protein for nucleotide binding folds and phosphoryl transfer catalysis [19,24,25]. The energetic role of adenylate kinase has gained particular significance following discovery that this enzyme facilitates transfer and utilization of γ- and β-phosphoryls in the ATP molecule through a chain of sequential reactions [1,26–28]. The adenylate kinase-catalyzed energy transfer shuttle and ligand conduction circuit concept [1,5,29–31] (Figure 1) is supported by biochemical, phosphoryl exchange measurements using ^18^Olabeling, physiological and gene-knockout studies [4,5,8,15,27–42], and is broadly used to explain energetic signaling mechanisms in heart and skeletal muscles [5,15,42–44], in hormone secretion [45–47], organ failure [4,31,48–50], tumor development [51–53], energy support of the cell nucleus [30,34,54,55], as well as in sperm and cell motility [8,56–59]. Muscles of AK1 knockout mice, with one less phosphotransfer chain, display lower energetic efficiency, slower relaxation kinetics and a faster drop in contractility upon ischemia associated with compromised myocardial-vascular crosstalk, AMP and adenosine generation, and impaired metabolic signal communication to the membrane metabolic sensor - the ATP-sensitive potassium channel (K-ATP) and distorted signaling to the energy-sensing AMP-activated protein kinase (AMPK) [4,5,1,32,33,35,39–42]. The unique ability of the ^18^O-assisted ^31^P-NMR technique to monitor adenylate kinase phosphotransfer and AMP signal dynamics in intact tissues, together with measurements of phosphotransfer rates through creatine kinase and glycolytic/glycogenolytic circuits and responses of metabolic sensors provide further insights into an integrated cellular energetic, metabolic monitoring and energy sensing interface [5,30,37,44,60].

Due to a unique property of the catalyzed reaction, adenylate kinase is recognized as a sensitive reporter of the cellular energy state, translating small changes in the balance between ATP and ADP into relatively large changes in AMP concentration [1,2,5,29,62]. This enables enzymes and metabolic sensors that are affected by AMP to respond with higher sensitivity and fidelity to stress signals [33,35,42,52,60–63]. Recent data further indicate that adenylate kinase mediated intracellular AMP signaling is coupled with a number of AMP-responsive elements including metabolic sensors, AMPsensitive metabolic enzymes and adenosine signaling [5,30,33,42,47,61–65]. By catalyzing nucleotide exchange and AMP signaling, adenylate kinase regulates the activity of glycolytic and glycogenolytic enzymes and provides an integrative node for both pathways to respond rapidly to fluctuating energy demands [5,30]. Adenylate kinase generated AMP is emerging as a potential metabolic signal whose intracellular and circulatory levels determine the balance of peripheral organ energy supply between glucose, glycogen and fat, thus regulating food intake, hormonal state, sleep, hibernation and body energy sensing in conjunction with hypothalamic AMP-activated protein kinase (AMPK), K-ATP channels and adenosine metabolic signaling cascades [4,5,30,63–69]. Thus, such integrated energetic and metabolic signaling roles place adenylate kinase as a hub within the metabolic regulatory system coordinating components of the cellular bioenergetics network.

A distinct role for adenylate kinase is emerging in cell motility, differentiation and mechanoelectrical signal transduction. Adenylate kinase has been implicated in spermatozoa motility by facilitating ATP delivery from midpiece mitochondria to remote ATPases in the tail [20]. It was demonstrated that through interaction with anchoring protein Oda5p adenylate kinase provides local ATP for dynein ATPase, ensuring that both high-energy phosphate bonds of ATP are efficiently utilized at the major site of power production of the microtubule motors involved in diverse cellular movements [57,70]. A recent study has demonstrated that a mutation in adenylate kinase 7 (AK7) underlies a primary ciliary dyskinesia phenotype in chronic obstructive pulmonary disease [7]. Moreover, van Horssen et al. [8] demonstrated that cytoskeleton-based cell motility can be modulated by spatial repositioning of adenylate kinase 1 (AK1) enzymatic activity providing local ATP supply and ‘on-site’ fueling of the actomyosin-machinery. More significantly and unexpectedly, mutations in the mitochondrial AK2 (adenylate kinase 2) gene have been identified in individuals affected with reticular dysgenesis, the most severe form of inborn severe combined immunodeficiencies (SCID), which is associated with sensorineural deafness, a process where nucleotide signaling, cell motility and ciliary functions are involved [9,10]. Knockdown of zebra fish *ak2* lead to aberrant leukocyte development, stressing the critical role of AK2, the major isoform in this type of cells, in leukocyte differentiation [10].

It is increasingly recognized that systemic integration of complementary energetic and metabolic signaling networks ensures cellular energy homeostasis and an adequate response to a broad range of functional activities and stress challenges [5,44]. A network and circuit view of the cellular energetic system allows for new perspectives leading to a comprehensive understanding of disease conditions associated with disturbances in energy metabolism, metabolic monitoring and signaling, and metabolic sensors response [5,71,72]. The purpose of this review is to summarize recent evidence regarding the role of the adenylate kinase phosphotransfer circuit in facilitating intracellular energetic communication, metabolic monitoring and AMP signal transduction. We highlight that due to intrinsic catalytic properties adenylate kinase is able to monitor and sense cell and body energetic imbalances caused by physical activity, inadequate oxygenation or nutrient supply. By generating and transmitting metabolic signals to a number of AMP/nucleotide-sensitive cellular and extracellular components, adenylate kinase is a primary player in adjusting cellular energetics, food intake, substrate transport and vascular blood flow to direct nutrient and oxygen delivery and maintain energy homeostasis.

## 2. Adenylate Kinase Isoform-Based Energetic and Metabolic Signaling Network

**Figure 2.**
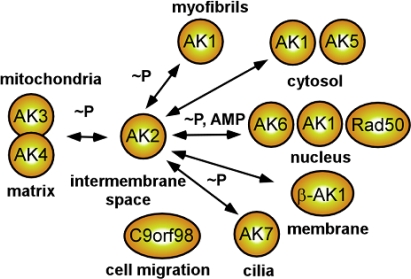
Adenylate kinase isoform network and intracellular localization. Adenylate kinase isoforms are coded by separate genes, KAD1 – KAD7, localized to different chromosomes. Corresponding proteins AK1 – AK7 define separate adenylate kinase isoforms with different molecular weights, kinetic properties and intracellular localization. The AK1 isoform mostly consists of the ADK domain which is characteristic for the whole protein family. The AK1β splice variant has an additional myristoylation domain that targets the protein to the plasma membrane. The proteins Rad50 and C9orf98 with ADK domains and activity have specific cellular functions. The AK2, AK3 and AK4 isoforms have a flexible lid domain which closes over the site of phosphoryl transfer upon ATP binding to prevent water accessibility. A short form of the lid domain exists also in AK1, AK5 and AK6. Within the network adenylate kinase proteins distribute high-energy phosphoryls (~ P) and communicate AMP signals.

The existence of multiple isoforms of an enzyme usually relates to their different intracellular distribution and kinetic properties according to the local metabolic requirements (Figure 2) [8,13,14,51,56,57,73]. Following the discovery of adenylate kinase more than six decades ago [see 57], three major isoforms, AK1, AK2 and AK3 have been identified, which are localized in the cytosol, mitochondrial intermembrane space and matrix, respectively [13,14,73]. They differ in molecular weight, structure, kinetic properties and nucleotide specificity [19,25,74]. AK1 and AK2 specifically bind AMP and favor binding to ATP over other nucleotide triphosphates, while AK3 is a GTP:AMP phosphotransferase specific for the phosphorylation of intramitochondrial AMP, but can only use GTP or ITP as a substrate [13,75]. Within the AK family there are several conserved regions, including a P-loop, AMP- and MgATP/MgADP-binding domains and a lid domain [19,25]. Adenylate kinase isoforms can form dimers and higher molecular order structures [76,77]. Phosphorylation of adenylate kinase has not been detected; but acetylation and myristoylation, which could facilitate binding of adenylate kinase to cell membranes, mitochondria or the nucleus, have been demonstrated [56,73,78]. Recently discovered glutathionylation of adenylate kinase could confer regulation of its activity by the cellular redox state [79].

Different polymorphic subforms of AK1 (AK1-1 and AK1-2) and splice variants of AK2 (AK2AD) have been found, which have distinct electrophoretic mobility and kinetic properties, i.e. AK2A (26.5 kDa) and AK2B (25.6 kDa) [2,5,80,81]. AK1-2 occurs only in the Caucasian populations and is common among hemophilia-A patients [81]. A recent study indicates that the negative effect of smoke on birth weight is more marked in AK1-1 mothers that in AK1-2 carriers, suggesting that zygotes carrying AK1-2 allele are more protected from damaging factors [82]. Interestingly, the specific activity of AK1-2 is about 3.5-times lower than that of AK1-1, whereas the Michaelis constants do not differ for the allelozymes [80]. Sequence analysis showed that Glu-123 in AK1-1 is exchanged for Gln-123 in AK1-2. Mitochondrial AK2-1(A) subform was found in about 30% of bipolar manic depression syndrome (MDS) patients, but in no case of unipolar MDS or controls. Also, tissue specific adenylate kinase isoforms AK4 and AK5 have been cloned which have mitochondrial matrix and cytosolic localization respectively (Figure 2) [2,23]. AK4 protein levels are increased in cultured cells exposed to hypoxia and in animal models of neurodegenerative diseases [83]. Although AK4 is enzymatically inactive it retains nucleotide binding capability, interacts with the mitochondrial ADP/ATP translocator and serves stress responsive protein function promoting cell survival and proliferation [83]. Both AK4 and AK3 are among hypoxia-inducible factor 1 (HIF-1) regulated genes promoting cell survival [84,85]. AK5 was detected in human pancreatic beta-cells and was implicated in regulation of the K-ATP channel [47], while appearance of autoantibodies to AK5 in refractory limbic encephalitis patients carries a poor prognosis [86]. More recently, the existence of an additional *AK1* gene product, the p53-inducible membrane-bound myristoylated AK1β, has been reported and implicated in p53-dependent cell-cycle arrest and nucleotide exchange in the submembrane space [51,73,87]. In this context, the gene encoding AK1 is down-regulated during tumor development, which could be associated with lower AK1β levels and cell cycle disturbances [88]. AK1β also has been demonstrated to be associated with the nuclear envelope [73] and proteomic studies have identified AK1β in epithelium microvilli [89], suggesting a role in energy support of nuclear and epithelia transport processes.

### 2.1. Adenylate kinase isoforms in the nucleus

The AK6 isoform was identified to be localized to the cell nucleus where energy provision and nucleotide channeling into DNA synthesis play a critical role in processing genetic information [34,54]. However, there is still controversy regarding the AK6 isoform which is also known as TAF9 RNA polymerase II possessing ATPase activity [90], suggesting that other adenylate kinase isoforms (AK1 and AK5) can also subserve nuclear energetic needs (Figure 2) [13,34,73]. Knockdown of AK6 slows growth and development of *C. elegans* [91], while in yeast a point mutation in Fap7 gene, an analog of AK6, reduces growth on glucose [92]. Another nuclear protein Rad50, a member of DNA repair RAD50/MRE11/NBS1 protein complex (RMN), which is essential for sensing and signaling from DNA double-strand breaks, in addition to ATP binding and hydrolysis bears an adenylate kinase activity required for efficient tethering between different DNA molecules [93]. A mutation affecting the adenylate kinase activity of Rad50, necessary for DNA tethering also abolishes the formation of viable spores [93]. Mutations in the genes that encode Nbs1 and Mre11, which are part of the RMN complex, are responsible for the human radiation sensitivity disorder, the Nijmegen breakage syndrome (NBS), and the ataxia-telangiectasia-like disorder (ATLD), which are characterized by defective checkpoint responses and high levels of chromosomal abnormalities [94]. Interestingly, the Nijmegen breakage syndrome gene product, Nbs1, is required for enzymatic activities of Rad50 and whole RMN complex function, including partial unwinding of a DNA duplex and efficient cleavage of fully paired hairpins [95]. Thus, adenylate kinase and adenylate kinase activity-possessing proteins play a significant role in the energetics of cell nucleus which is separated from major ATP generating processes in the cytosol.

### 2.2. Adenylate kinase isoforms in cell motility and nucleotide pool homeostasis

The large molecular weight isoform AK7 is associated with cell motility and other processes [5]. Recently, a high level of expression of the AK7 isoform has been demonstrated in bronchial epithelium and appears to be associated with ciliary function [96]. AK7 is a differentiation marker of kinocilia-bearing cells [97], and mutation in the AK7 gene is associated with human ciliary dyskinesia [7]. In this regard, Foxj1, a forkhead transcription factor necessary for ciliogenesis, induces adenylate kinase isoforms AK5 and AK7 along with genes whose products comprise dynein arms [98]. Yet to be named, a protein with adenylate kinase domains coded by chromosome 9 open reading frame 98 (C9orf98) gene is associated with cell migration and nucleotide exchange [99]. In this regard, sea urchin embryo cilia and sperm flagella use high-molecular weight adenylate kinase with triplicated catalytic domains to power swim by ciliary and flagellar movement [100]. Thus, multiple adenylate kinase isoforms create a phosphotransfer network to serve specific needs in different cellular compartments for energetic and metabolic signaling.

Heart muscle harbors about 30 – 40% of adenylate kinase activity in mitochondria particularly in the intermembrane space [26,74]. The mitochondrial AK2 isoform has the highest affinity (lowest Km) for AMP (≤ 10 μM) among AMP metabolizing enzymes and is highly concentrated in the narrow intermembrane space [18,26,74]. Virtually all the AMP reaching mitochondria is converted to ADP and channeled into oxidative phosphorylation maintaining a low cytosolic AMP concentration [21,35,41,101]. In such a way, adenylate kinase tunes cytosolic AMP signals and guards the cellular adenine nucleotide pool [1,41,102,103]. During intense physical activity or metabolic stress, such as ischemia, the AMP concentration rises, turning on other AMP-metabolizing enzymes such as AMPdeaminase and 5’-nucleotidase producing IMP and adenosine [60,104]. In this regard, a marked elevation of mitochondrial AK2 activity has been demonstrated in hypertrophy in response to increased energy demand and the necessity to maintain the cellular adenine nucleotide pool [105]. In Drosophila, AK2 is essential for survival and circadian rhythm formation and the lack of the AK2 gene causes significant insect growth suppression [2]. AK2 is downregulated in Keloids disease associated with fibroproliferative dermal tumors developing as a result of deregulated wound healing [106].

Expression of adenylate kinase isoforms increases in response to muscle exercise, hypoxia, and metabolic stress [107,108]. Also, muscle exercise performance correlates with adenylate kinase activity, signifying that this enzyme is an integral part of cellular energetic homeostasis [108]. In addition, a decreased expression and activity of AK1 has been found in a mouse model for muscular dystrophy (mdx mice) suggesting a direct relationship between lack of dystrophin and alteration of AK1 inducing energetic deficit [109]. A significant increase in AK1 in obese patients could indicate an imbalance in AMP signaling in this metabolic syndrome [110].

### 2.3. Adenylate kinase localization and interacting partners

Although major compartments for adenylate kinase isoform localization are known based on cellular fractionation, intimate intracellular anatomy of adenylate kinase distribution has only been studied by immunocytochemistry in neuronal cells and skeletal muscle [74,111,112]. In skeletal muscle myofibrils, adenylate kinase is localized in linear arrays along with creatine kinase and glycolytic enzymes [111]. Similar localization of GFP-tagged AK1 was detected in neonatal cardiomyocytes [76]. Such sequential arrangement of adenylate kinase molecules could provide a bidirectional phosphorelay that links ATP-generation with ATP-consumption and ATP-sensing processes [1,5]. Another study using tagged proteins indicates that AK1β-EGFP is mainly localized on the plasma membrane, whereas AK1-EGFP is distributed throughout the cell except for trace amounts in the nuclear membrane and some vesicles [87]. Adenylate kinase was found to co-purify and presumably interact with glycolytic enzymes and associate with myofibrils, cellular and mitochondrial membranes [21,26,33,51,73,74,78,113]. Adenylate kinase was also found to be engaged in intimate functional/structural interactions with the sarcolemmal K_ATP_ channel, a major metabolic sensor [33,46,47]. More recently, a phosphotransfer enzyme anchoring protein FHL2 was discovered in heart muscle [76]. This protein positions adenylate kinase and other phosphotransfer enzymes close to ATP utilization sites in myofibrils ensuring that both high-energy phosphate bonds of ATP are efficiently utilized. Mutation of the FHL2 protein is associated with cardiomyopathy [76]. Another adenylate kinase anchoring protein, Oda5p, anchors adenylate kinase in the proximity of the dynein arm ensuring that both high-energy phosphate bonds of ATP are efficiently utilized at the major site of power production of the microtubule motors involved in diverse cellular movements [57,70]. In epididymal spermatozoa adenylate kinase AK1 interacts with sperm associated protein P25b within cholesteroland sphingolipid-enriched membrane domains [114]. Such association and localization is necessary for peri-membrane space ATP homeostasis and for sperm maturation and fertilization [114].

A direct interaction between adenylate kinase and several enzymes of the dNTP synthase complex, as well as nucleoside diphosphate kinase was demonstrated using protein affinity chromatography and immunoprecipitation [115]. These results identified adenylate kinase as a specific component of the nuclear dNTP synthase complex, where it facilitates the survey of nucleotide ratios and the synthesis of nucleotides necessary for error-free DNA replication. Although topological positioning of specific proteins is crucial in energetics and signaling processes, the full interactome of adenylate kinase isoforms in cardiac muscle and other tissues is still unknown. In summary, molecular diversity and intracellular arrangement of adenylate kinase isoforms serve as a prototype of energetic and metabolic monitoring network where the localization of enzymes, their kinetic properties and the ability to communicate between different compartments play a critical role.

## 3. Adenylate Kinase Catalyzed β-Phosphoryl Transfer and Energy Economy

Adenylate kinase’s unique energetic function allows the utilization of the second high-energy bond of the β-phosphoryl in the ATP molecule, thereby doubling the energetic potential, a property not shared by other phosphotransfer systems [1,5,12,20,26,29]. This catalytic function of adenylate kinase is particularly important in tissues with high and fluctuating energy demands, as well as those under metabolic stress [1,2,5]. It is also important for the intimate energy supply to specific cellular processes such as cell ciliary, flagella or cytoskeleton based motility [8,20,29–31]. A concerted action of both cytosolic and mitochondrial adenylate kinase isoforms is required to facilitate high-energy phosphoryl delivery to cellular ATPases and feedback signal communication to mitochondrial respiration within structurally organized enzymatic modules and networks (see Figure 1) [1,26,29,31]. In these networks, a series of rapidly equilibrating reactions catalyzed by adenylate kinase provide the driving force for high-energy phosphoryl flux [1,5,30]. In addition, adenylate kinase coupled with creatine kinase and glycolytic pathways communicate adenine nucleotide flux changes generated by cellular ATPases to metabolic sensors [5,29,33,37]. In such a way phosphotransfer reactions synchronize electrical and mechanical activities with energy supply processes, which is fundamental for optimal function of the heart [44,116]. Adenylate kinase is one of principle components in the generation of metabolic oscillations by sustaining dynamic fluctuations of adenine nucleotide ratios [5,29,117,118]. In this regard, the term of excitable “adenylate kinase medium” has been proposed [119] to emphasize the significance of this enzyme in conveying energetic and metabolic signals [5,33]. These functions render adenylate kinase essential to the integrated cellular phosphotransfer network sustaining an efficient and vibrant cell energetic economy.

Further insights and key support for the current understanding of metabolic signaling networks in their full complexity have come with the development of new methodologies [1,4,15,31,44,60]. Highenergy phosphoryl fluxes through adenylate kinase, captured with ^18^O-assisted ^31^P-NMR, tightly correlated with the performance of the myocardium under various conditions of stress load [120]. This implicates that adenylate kinase along with other phosphotransfer reactions are indispensable routes that direct flow of high-energy phosphoryls between cellular ATPases and the ATP production machinery in mitochondria [44]. Labeling studies indicate that in intact tissues the highest adenylate kinase-catalyzed β-phosphoryl phosphotransfer flux is in the kidney, which approximates 98% of γ-ATP turnover, followed by the liver (80%), the heart (15 – 22%) and contracting (10 – 17%) or resting (3 – 5%) skeletal muscles suggesting an important role of adenylate kinase in tissue energy homeostasis [5,30,41]. Specifically, the total adenylate kinase enzymatic capacity measured in vitro in murine heart is about 6 mM/s, while the phosphotransfer flux in vivo is between 0.2–0.3 mM/s, it increases with functional load and can reach 1 mM/s in hypoxia and is expected to rise even more in ischemia [5]. For comparison, the range of phosphotransfer capacities of creatine kinase and glycolytic pathways are 6–10 mM/s and 2–3 mM/s, respectively [5]. In this regard, activities of adenylate kinase isoforms and intracellular free AMP levels tightly correlate with tissue respiration rates [16,121] and expression of adenylate kinase isoforms increases in response to muscle exercise, hypoxia, and metabolic stress [107,108]. Indeed, adenylate kinase remains active in its ATP regenerating and transferring role as long as ADP is available and the enzyme is not inhibited by a build-up of AMP [12,13]. Adenylate kinase phosphotransfer flux is markedly suppressed by high glucose in insulin secreting cells, reducing adenylate kinase-mediated AMP signaling to the K-ATP channel and AMPK, two key regulators of hormone secretion [45,122]. There is a reciprocal compensatory relationship between adenylate kinase and creatine kinase phosphotransfers to safeguard cellular energy economy: reduction of creatine kinase activity promotes high-energy phosphoryl transfer through the adenylate kinase system in creatine kinase-knockout muscles or under hypoxic stress [1,15,28,36,60].

The condensed mitochondrial structures and a very narrow intracristal space in the living cell poses diffusional limitations for nucleotide exchange [29]. Adenylate kinase (AK2) in the intermembrane space appears necessary to conduct the ADP stimulatory signal through the adenine nucleotide translocator to matrix ATP-synthases, as well as in exporting ATP produced by oxidative phosphorylation [2,21,29]. Disruption of the adenylate kinase gene impedes ATP export and mitochondria-cytosolic communication in yeast [101]. Muscles of AK1 knockout mice display lower energetic efficiency, slower relaxation kinetics and can not sustain low ADP levels under a functional load despite the presence of active creatine kinase, mitochondrial oxidative phosphorylation and glycolytic/glycogenolytic ATP-regenerating pathways, indicating disruption of the coherent energetic network with blunted response to metabolic signals [15,40–42]. Genetic disruption of both cytosolic M-CK- and AK1-catalysed phosphotransfer pathways compromises intracellular metabolic communication and energetic efficiency, reducing the cellular capability to maintain total ATP turnover under functional load [39]. These new methodologies and transgenic gene manipulations provide the opportunity to decipher regulatory mechanisms that underlie cardiac and skeletal muscle bioenergetic homeostasis.

Taken together, studies of adenylate kinase gene-knockout models have opened new perspectives for the further understanding of how cellular energetic and metabolic signaling networks integrate with genetic, biosynthetic, membrane-electrical and receptor-mediated signal transduction events [2,5,32,33,35,39]. Gene-knockout studies also revealed a remarkable plasticity of the cellular phosphotransfer system, where deficiency in an individual enzyme is compensated through the remodeling of the whole energetic network at enzymatic, architectural and genomic levels [15,36,123,124]. Unexpectedly and contrary to common beliefs that adenylate kinase promotes nucleotide degradation, the AK1 deficient heart had less ability to maintain nucleotide pools under metabolic stress [32,35]. Also, in failing hearts adenylate kinase activity tightly correlates with higher cellular adenine nucleotide content [49,50], indicating a new function for adenylate kinase to safeguard the cellular nucleotide pool by rephosphorylating AMP back to ADP and ATP.

Emphasizing the significance of intact energetic and metabolic signaling, AK1 deficiency is associated with a range of compensatory changes in glycolytic, glycogenolytic and mitochondrial metabolism and corresponding gene expression to support energy metabolism [4,15,32,35,39,123]. In this regard, one of the major finding resulting from adenylate kinase and creatine kinase knockout studies was the discovery that glycolytic/glycogenolytic enzymes have the ability to provide a network capacity for transferring and distributing high-energy phosphoryls [1,5,36]. The function of the adaptor protein DRAL/FHL-2, which anchors adenylate kinase, creatine kinase and glycolytic enzymes to sites of high energy consumption in myofibrils [76], further highlights the significance of the topological arrangement and integration of the intracellular phosphotransfer network in matching cellular energetic needs.

In summary, adenylate kinase-facilitated high energy phosphoryl transfer and coordination between cellular sites of ATP consumption and ATP generation are essential for the safeguard of the cellular nucleotide pool and energy economy. Although significant progress has been made, there are still significant unanswered questions concerning adenylate kinase physiology, especially regarding the energetic role of mitochondrial intermembrane AK2 and matrix AK3, nuclear AK6, ciliary AK7 and other tissue specific adenylate kinase isoforms.

## 4. Adenylate Kinase and AMP Signaling: An Integrated Metabolic Monitoring and Signal Communication System

**Figure 3.**
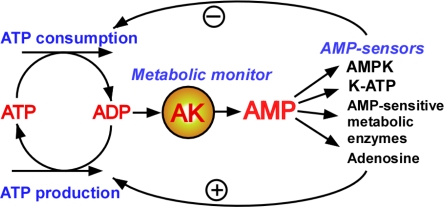
Adenylate kinase metabolic monitoring system. Adenylate kinase reads the cellular energy state, generates, tunes and communicates AMP signals to metabolic sensors. In such way adenylate kinase conveys information about the adenine nucleotide pool status and, thus, the overall energy balance. In response to AMP signals metabolic sensors reduce ATP-consuming and activate ATP-generating pathways to adjust energy metabolism, functional activity and increase fuel and oxygen supply.

Growing evidence indicates the significance of metabolic monitors which directly sense cellular energy state and respond to metabolic imbalances by generating and delivering signaling molecules to metabolic sensors and effectors to produce a regulatory response [1,2,5,52,62]. Central in metabolic monitoring is the enzyme adenylate kinase which constantly reads cellular adenine nucleotide balance, and, in case of disparity, generates AMP signals and facilitates their delivery to a number of AMPsensitive components, including those in the gycolytic and glycogenolytic pathways and to metabolic sensors and effectors such as K-ATP channels and AMPK, which adjust tissue energy and body hormonal state (Figure 3) [5,29,37,61]. Both K-ATP channels and AMPK can regulate cellular energy balance through managing Ca^2+^ influx and by phosphorylating targeted proteins [5,44]. Due to a unique property of the adenylate kinase-catalyzed reaction, a small decrease in ATP levels results in a large increase in AMP, making the latter a sensitive indicator and therefore a suitable signaling molecule of cellular energetic status [1,5,62]. In recent years AMP signaling is emerging as one of the most versatile system in the regulation of diverse cellular processes [5,72]. Importantly, AMP signals must be integrated and tuned to an appropriate level, since low or excess AMP signaling, due to metabolic, hormonal state or metabolic sensors mutations, are associated with disease conditions [5,29,37,61,62,72,125]. As a result of adenylate kinase-mediated metabolic monitoring and AMP signaling (Figure 3), the activity of ATP generating pathways is increased while ATP-consumption is decreased.

In particular, adenylate kinase phosphotransfer directly couples with K-ATP channels, facilitating the translation of metabolic signals critical in adjusting cellular excitability-dependent functions in response to demand [1,33,37,46,47,61]. In the intracellular environment where diffusion is restricted, reactions tend to depend strongly on the local rather than global concentrations of metabolites [37]. In this way, adenylate kinase-catalyzed AMP signal generation and nucleotide exchange in the intimate “sensing zone” of metabolic sensors regulate the dynamics and frequency of ligand switching in order to facilitate decoding of cellular information [5,30,44]. Indeed, intracellular measurements using the ^18^O-assisted ^31^P NMR and mass spectrometric techniques indicate that adenylate kinase is uniquely situated to tune the magnitude of the AMP signal because its phosphotransfer displays only a fraction of total capacity, is compartmentalized and not universally at equilibrium, thus it can differentially promote both AMP signal generation and AMP rephosphorylation [1,4,28,31,36,38,45,60,120,126]. In this way, adenylate kinase through negative and positive feedback loops governs adenine nucleotide and glycolytic oscillations providing a dynamic component for facilitated intracellular energetic signal communication [5,29,117,119]. Moreover, through a series of spatially linked enzymatic reactions adenylate kinase facilitates propagation of nucleotide signals in the intracellular and extracellular space, thus coordinating the response of metabolic sensors and nucleotide/nucleoside receptor signaling [1,5,64,65]. Therefore adenylate kinase provides sustained communication between cytosolic and nuclear processes coordinating cell energetic and genomic events [30,34] and between myocardium and vasculature regulating coronary flow and oxygen delivery [4].

Adenylate kinase-induced increase in AMP promotes the generation of adenosine, a powerful metabolic signaling and cardioprotective agent [60,104]. AMP also triggers AMPK activation, an essential signaling module in cellular adaptation to stress [42,52,62,127]. In the heart, the significance of AMPK is suggested by the findings that AMPK activity is increased in ischemia and preconditioning, and that AMPK agonists protect myocardium against ischemic injury [127,128]. Hypoxia or metabolic stress increases adenylate kinase flux inducing AMP generation and downstream AMP/adenosine signaling events important in cardioprotection [1,59,63,104]. Adenylate kinase deficiency compromises metabolic signal reception by metabolic sensors, such as K-ATP channels and AMPK producing a stress vulnerable phenotype [33,42]. In this regard, new data suggests that hydrolysis of AMP by CD73 on graft-resident or circulating cells regulates endothelial barrier function and diminishes transendothelial leukocyte trafficking and mitigates inflammatory and immune response of cardiac transplantation via the A(2B) adenosine receptors [129]. Thus, adenylate kinasemediated AMP signaling through metabolic sensors and nucleoside receptors is an integral part of cellular stress response system.

Adenylate kinase does not act alone in performing cellular metabolic monitoring. Recent evidence indicates that the interaction between adenylate kinase and creatine kinase phosphorelays determines metabolic signal communication to the K-ATP channel and mediates energetic remodeling in preconditioned and failing hearts [29,33,34,37,41,44,116]. Under normal conditions creatine kinase suppresses adenylate kinase phosphotransfer by scavenging cellular ADP [28] and thus maintaining the K-ATP channels in the closed state with low metabolic signaling through the AK → AMP → AMPK and AK → AMP → adenosine axis [33,52,61,105,116]. However, hypoxia or metabolic stress diminishes creatine kinase and increases adenylate kinase flux inducing AMP generation and downstream AMP/adenosine signaling events to adjust cellular energy balance [1,60,63,126]. Indeed, deletion of the AK1 gene shifts this balance towards the creatine kinase system, compromising energetic signal communication [4,33,35,39]. Accordingly, AK1 deficiency blunts metabolic signal reception by metabolic sensors, such as K-ATP channels and AMPK, compromising their ability to withstand energetic stress [33,42]. Underscoring the significance of phosphotransfer redistribution in metabolic signaling is the observation that altered adenylate kinase and creatine kinase phosphotransfer enzyme activities are associated with hypertrophy, abnormal vascular tone and hypertension [105,130,131].

The role of adenylate kinase in integrating signaling pathways is further indicated by the recent demonstration that AMP-stimulated AMPK regulates vascular response to hypoxia and nitric oxidedependent vasorelaxation as well as excitation of the oxygen-sensing carotid body which is critical for detection of O_2_ deficit in the bloodstream and adjustment of breathing pattern [132–134]. In addition, adenylate kinase appears to be the major phosphotransfer system in extraocular muscle, where, together with creatine kinase, it regulates precise eye movements [135]. A more recent study suggests that intracellular and extracellular adenylate kinase plays an important role in nucleotide energetic signaling regulating actin assembly-disassembly involved in cell movement and chemotaxis [136]. Thus, the balance between phosphotransfer systems and subsequent signaling events determine the outcome of metabolic regulation of muscle contractility, electrical activity and vascular tone.

In recent years, adenylate kinase-mediated extracellular AMP and ADP signaling has gain an additional significance due to the involvement in high-density lipoprotein (HDL) endocytosis, in signaling through adenosine and AMP-specific receptors [137] and through AMP and other metabolite-sensing RNA riboswitches [138, 139], as well as in cell differentiation, tumor suppression and regulation of vascular tone [5,30,45,51,53,55,64,65,140,141]. In this regard, metabolic monitoring system in procaryotic cells utilizes RNA structures embedded at the 5′ ends of mRNAs to sense particular metabolic cues and regulate the encoded genes [142]. By sensing the energy status of muscle cells and regulating gene expression, the adenylate kinase and downstream AMP signaling are critical regulators of mitochondrial itochondrial biogenesis, through the AMPK-PGC-1 signaling cascade, thereby increasing the energy transducing capacity of the cell [138,143]. Moreover, adenylate kinase through AMP signaling regulates glycolytic and glycogenolytic pathways conveying information regarding increased energy demand [5]. Regulation of glycogen metabolism is of primary importance in muscle energetics, including that of the heart [5,144]. Particularly, defective AMP and AMPK signaling is a primary cause of imbalance in glycogen metabolism and associated disease conditions [145]. Adenylate kinase metabolic monitoring plays a significant role in plant tissues [146]. Modulation of plastidial adenylate kinase activity and consequently AMP and adenine nucleotide levels significantly increases starch yield and amino acid biosynthesis [147,148], while overexpression of adenylate kinase in yeasts markedly increases ATP production [149]. Thus, adenylate kinase by providing ATP β-phosphoryls for energetic needs and by regulating nucleotide ratios and AMP signaling adjusts the efficiency of energy metabolism, the capacity of ATP generating reactions and the activity of biosynthetic processes.

Intracellular energetic and metabolic signal communication can employ several different mechanisms ranging from facilitated diffusion to ligand conduction, depending on cell type and specific compartment, structural organization and topological arrangement of enzymatic networks [29,30]. While spatial heterogeneity and directionality of the enzyme-catalyzed process is not important in well mixed conditions *in vitro,* this becomes very important entity in highly organized living matter [150]. The cluster organization of enzymes and the high rate of unidirectional phosphoryl exchange in phosphotransfer systems would promote ligand conduction and signal communication at cellular distances [1,29,30]. Adenylate kinase facilitates mitochondria-nuclear energetic communication [34] and within the nucleus, embedded into organized enzymatic complexes of nucleotide-metabolizing and phosphotransfer enzymes, is involved in maintaining proper nuclear dNTP ratios and facilitates channeling of nucleotides into DNA replication machinery [115]. Imbalances in dNTP ratios affect the fidelity of DNA replication and repair leading to acquired mutations [30,115]. Thus, adenylate kinase surveys nucleotide ratios necessary for error-free DNA replication, while another nuclear protein Rad50, harboring adenylate kinase activity, participates in DNA double-strand break repair [93].

Despite recent advances in the metabolic signaling field, more studies are necessary to elucidate mechanisms which link adenylate kinase phosphotransfer flux, AMP signal dynamics and the response of metabolic sensors (AMPK, K-ATP), as well as the significance of AK → AMP → AMP-sensors signaling system in heart physiology, such as in shaping heart force-frequency relationship and Frank- Starling response, and cardioprotection [5,44]. At the molecular level, studies of multienzyme complexes including adenylate kinase would shed light on intimate mechanisms of metabolic sensing. Co-localization of components in signal transduction cascades is critical for the directionality and specificity of the signaling response. The presence of adenylate kinase in close proximity of AMPK would facilitate metabolic signal transduction and create a favorable energetic environment for the phosphorylation of targeted proteins. The significance and downstream mechanisms by which AK → AMP → AMPK signaling axis regulate cell cycle, developmental potential, and timing of differentiation are just starting to be elucidated. Thus, although more studies are needed, adenylate kinase-mediated metabolic monitoring and downstream AMP signaling is increasingly recognized among major homeostatic mechanisms in a number of cells, critical in the regulation of diverse cellular processes and stress-response.

## 5. AMP as Universal Fuel Consumption and Low Energy Signal, Mediator of Drug Action and Therapeutic Agent

**Figure 4.**
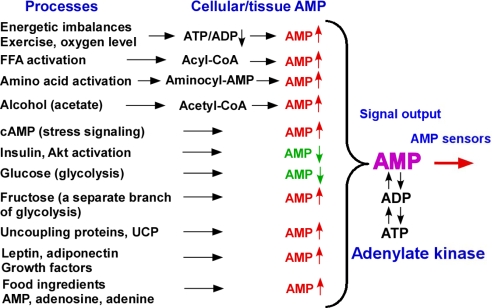
AMPing up and down — integration cellular AMP signals by adenylate kinase. Adenylate kinase integrates AMP metabolic signals produced or downregulated during exercise, stress response, food consumption and during changes in hormonal balance or mitochondrial coupling state. Adenylate kinase relays deliver AMP signals to metabolic sensors and by catalyzing nucleotide exchange in the intimate “sensing zone” of metabolic sensors facilitate decoding of cellular information.

Metabolic signals regulate and integrate many vital functions throughout the human body, including energy homeostasis, blood pressure, heart performance, food intake, hormonal status and brain performance [30,71]. Recent evidence suggests the general importance of adenylate kinase-mediated AMP signaling in appetite, wakefulness and sleep control and in hormonal, food and antidiabetic drug actions which are coupled to alterations of cellular AMP levels and associated signaling [4,5,46,47,55,62–70,86,138,151]. AMP signaling also plays an important role in hypoxic response, immune function and taste reception [60,85,129,137,139]. Adenylate kinase integrates and tunes AMP signals coming from different sources and delivers them to metabolic sensors to elicit a regulatory response (Figure 4). Reduced or increased adenylate kinase activity would distort integration of AMP signals and, depending on tissue metabolic phenotype and intensity of AMP-generating reactions, could produce both positive and negative impact on the activity of metabolic sensors [42,152].

Recent evidence suggests that ingestion of fructose, a major constituent of sugar and high-fructose corn syrup, causes increase in cellular AMP and AMP/ATP ratio leading to activation of AMPK [68, 153]. Contrary to glucose, which usually lowers cellular AMP levels and inhibits AMPK, fructose activates hypothalamic AMPK and increases food consumption (Figure 4) [68]. Compared with glucose-sweetened beverages, consumption of fructose-sweetened beverages with meals elevates postprandial plasma triglycerides and lowers 24-h insulin and leptin profiles in normal weight women [154]. Fructose metabolism bypasses the rate-limiting step of the glucose pathway, and is metabolized much more quickly than glucose. Excessive fructose intake depletes cellular ATP by trapping inorganic phosphate required for ATP resynthesis, consequently inducing nucleotide degradation and increasing plasma uric acid and allantoin levels [155]. These metabolic alterations induced by fructose may play an important role in the epidemic of metabolic syndrome and may contribute the development of diabetes and cardiovascular disease [156]. A diet high in fructose can give rise to hyperlipidemia, insulin resistance and hypertension [157]. Similarly, ethanol consumption and subsequent acetate metabolism causes AMP accumulation in liver and other tissues resulting in increased AMP and adenosine signaling and blood flow [158,159]. Excess ethanol consumption can also cause nucleotide degradation and depletion of cellular adenine nucleotide pool [160]. However, short term and limited exposure to fructose and ethanol could be beneficial in stimulating energy metabolism and metabolic signaling, thus “AMPing” your body [153,161]. In this regard, cardioprotection induced by both fructose and ethanol preconditioning stimuli could be related to augmented AMP signaling [162,163].

**Figure 5.**
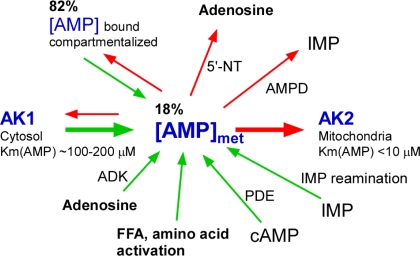
Regulation of intracellular AMP levels. Cytosolic adenylate kinase (AK1) is the major AMP generator while mitochondrial AK2 isoform, due to low Km(AMP), is the major AMP sequestration and tune-up mechanism. AMP is also generated during free fatty and amino acid activation, during adenosine rephosphorylation by adenosine kinase (ADK), during IMP reamination and by cyclic nucleotide phosphodiseterase (PDE). Oxygen deprivation and intense muscle contraction increase AMP removal through adenosine and IMP pathways catalyzed by 5’-nucleotidase (5’-NT) and AMP-deaminase (AMPD). Defects in mitochondria metabolism would reduce AMP tuning capacity of AK2 and, in fact, can reverse reaction towards AMP generation. The metabolically active AMP pool, estimated about 10–20%, is in dynamic equilibrium with bound and/or compartmentalized AMP [1,5].

Normal human blood AMP levels are in the 10–20 μM range of which only a fraction is free due to binding to serum proteins [164]. Intracellular and blood AMP levels are increased during physical activity and are sensitive indicators of myocardial ischemia rising within minutes after insult [165,166]. Adenylate kinase isoforms provide fine tuning of intracellular, interstitial and circulating AMP levels due to different kinetic properties and localization (Figure 5). Deficiency of AK1 isoform reduces the AMP signal stress response [4,32,33,35,42], however at the basal level it could result in higher AMP signaling due to compromised tune-up mechanisms [152]. Circulating AMP is emerging as a molecular mediator of hibernation and constant darkness effect, switching mice from a glucoseburning, fat-storing state to a fat-burning, glucose-conserving lethargy [66]. In hibernating mammals AMP originating, at least partly, from the brown fat also down-regulates the seasonally-dependent proliferation of the thymus [167]. In addition, overworked brains release adenosine, usually originating from AMP, to slow cell activity and trigger sleep [168,169]. These data strongly support the notion that AMP and adenosine play a key role as endogenous modulators of wakefulness and sleep which fits with our proposed role of phosphotransfer reactions in regulating brain activity and information processing [30].

Available evidence suggests that growth factors, which alter cellular energy metabolism, and hormones, such as adipocyte-derived leptin and adiponectin, activate AMPK through local or temporal regulation of AMP levels [170–172]. Leptin alters muscle adenine nucleotide homeostasis (decreases ATP) and increases AMP dynamics (inferred from elevated AMP and IMP levels) followed by activation of AMPK [173–175]. This could be also due to increased expression of uncoupling proteins (UCP) - mitochondrial carriers that dissipate the electrochemical gradient across the mitochondrial inner membrane [176,177]. Mild uncoupling of mitochondria shift cellular nucleotide balance and, due to the monitoring function of adenylate kinase, AMP levels would go up triggering metabolic signaling cascades [176,177]. Interestingly, AMP by itself through interaction with ANT can produce uncoupling that could facilitate respiration and reduce ROS production [178], which could be beneficial during ischemia reperfusion.

AMP signaling plays a significant role in cellular senescence. It’s been shown by proteome analysis that AK1 protein is markedly increased in senescent skeletal muscle fibers [179] and that lifespan of worms can be extended by the addition of copies of the AMPK gene and by chronic activation of AMPK as is seen on calorie-restricted diets [180]. Indeed, AMP/ATP ratios are several folds higher in senescent fibroblasts compared with young fibroblasts and this is accompanied by a marked elevation in AMPK activity [181,182]. This could be viewed as a compensatory measure to cope with declining capacity of energy metabolism during aging. AMP and AMPK signaling is critical in cell differentiation, maintaining cell polarity and completing normal cell division as well as in induction of meiotic maturation in mammalian oocytes [183–185]. AMP directly or through AMPK plays a physiological role in modulating activity of cystic fibrosis transmembrane conductance regulator (CFTR) in polarized epithelia cells [186,187]. CFTP nucleotide binding folds possess an intrinsic adenylate kinase activity which could facilitate metabolic signal reception [61,188]. Thus, AMP signaling plays a critical role in altered hormonal and energetic states including cell differentiation, maintenance of polarity and senescence.

AMP is important mediator of insulin and metabolic protein kinase Akt signaling. It has been proposed that protein kinase Akt mediates inhibitory effects of insulin on AMPK [189]. Since Akt does not directly phosphorylate AMPK, changes in Akt activity induced by insulin can regulate AMPK through changes in cellular AMP/ATP ratio. Indeed, it was demonstrated that Akt activation reduces cellular AMP/ATP ratio causing decline in AMPK activity [190]. Insulin and Akt-mediated inhibition of AMPK can be overcome by metformin, which is known to act on site I of mitochondrial respiratory chain causing increase in AMP levels [189,191]. In this regard, free fatty acids, which activation generates AMP, prevents AMPK inhibition by insulin [192]. Thus, insulin-Akt signaling axis can expand the range of metabolic effects through tuning up AMP signals and the activity of AMPK.

Most importantly, recent studies indicate that pharmacological actions of popular antidiabetic drugs metformin and thiazolidinediones and cholesterol lowering statins are related to their ability to alter cellular AMP levels and consequently AMPK activity [151,193–195]. Time course studies revealed that troglitazone-induced increases in phosphorylated forms of AMPK and ACC are paralleled by an increase in the AMP-to-ATP ratio [193]. A similar increase in AMP is seen following incubation of cells with rosiglitazone [196]. Moreover, livers of rats treated with resveratrol, a constituent of red grapes and red wine, show a strong tendency for AMPK activation, as well as increase phosphorylation of two downstream indicators of its activity [197]. Besides inhibiting HMG-CoA reductase, statins preserve CD39/ATPDase activity in thrombin-treated endothelial cells [198]. Preservation of adenine nucleotide metabolism may directly contribute to the observed anti-thrombotic and anti-inflammatory actions of statins. In this regard, metformin, a biguanidine compound from French lilac and more recently extracts from bitter melon, a traditional Chinese medicine, activate AMPK and exerts its glucose lowering effect through mild interference with the efficiency of energy metabolism resulting in changes in intracellular nucleotide dynamics and AMP levels [151,194,199]. Through activating AMP signaling metformin also improves cardiac function after ischemia in rats [200, 201]. Interestingly that metformin increases glucose utilization and lactate production in cells with a dominant-negative mutant form of AMPK (DN-AMPK) [202], suggesting existence of AMPKindependent pathways of metabolic signaling including direct effects of AMP on other metabolic sensors and enzymes of energetic pathways.

Recent interest in clinical use of adenosine as “adenosine flush” and in reperfusion therapy is also associated with AMP signaling, activation of AMPK and replenishment of cellular ATP levels [203–205]. Adenosine, besides signaling through adenosine receptors, can be taken up by cells and phosphorylated to AMP initiating metabolic signaling cascades [104,206]. Subsequent conversion of AMP to ADP and ATP by reactions involving adenylate kinase and ATP synthesis in mitochondrial oxidative phosphorylation and glycolysis would replenish cellular ATP and total adenine nucleotide pool which is diminished during ischemic insult [104,206]. Thus, adenosine and AMP have pleiotropic metabolic signaling and energetic effects which could be further explored in reperfusion therapy.

Last but not least, AMP through interaction with taste receptors has the bitterness-suppressing quality that allows taste buds to interpret food as seeming “sweeter” [207]. This makes lower-calorie food products more palatable. Recently AMP has been approved by the FDA as a ‘Bitter Blocker’ additive to foodstuffs (Linguagen Corp.). AMP is found in a wide range of natural foods — including breast milk. Calcium compounds in breast milk are usually bitter and thus breast milk may be natural system using bitter-blockers. AMP also inhibits behavioral and electrophysiological responses of mice to bitter tastants [207]. A number of AMP containing medications are used for nutritional supplementation and for treating dietary shortage or imbalance and disease conditions [208]. Nucleotides such as AMP affect a number of immune functions, including the reversal of malnutrition and starvation-induced immunosuppression due to adenine nucleotide shortage, the enhancement of Tcell maturation and function and natural killer cell activity [208]. However AMP by itself has immunosuppressive properties. In fact, mosquito and fly saliva contains AMP and adenosine as vasodilatative agents which also have immunosuppressant activity facilitating pathogen or parasite transmission [209]. To this end, secreted and cell associated adenylate kinase has been identified as a virulence factor in a number of pathogens affecting nucleotide signaling and host immune defense [210]. Due to immunomodulatory function, a promising therapeutic potential for the AMP analog AICAR/(ZMP) exist in the treatment of multiple sclerosis and other Th1 cell-mediated inflammatory diseases such as psoriasis and arthritis [211]. Thus, AMP is emerging as pivotal metabolic signal conveying information about food consumption, hormonal and energy metabolism status, a regulator of brain activity associated with wakefulness and appetite control as well as a mediator of drug action and therapeutic agent.

## 6. Adenylate Kinase and AMP Signaling Networks in Body Energy Sensing

Sensing body energy level and corresponding mental and physical strength is important for humans and animals and this property had key survival and evolutionary advantages [5,30,72,212–216]. Here we further advance the hypothesis that body energy sensing is mediated in part by adenylate kinase, which conveys information about the adenine nucleotide pool status and, thus the overall energy balance [5,30]. The general concept has been proposed previously by H. Teagtmeyer [217] that systemic metabolic communication and energy transfer arises from the interaction of a series of moiety conserved cycles operating through tissues and circulation.

**Figure 6.**
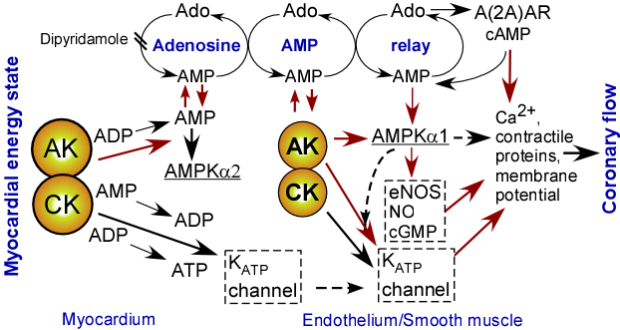
Myocardial-vascular metabolic signaling as a paradigm of global energy sensing. Metabolic signal transduction cascades initiated by phosphotransfer redistribution between adenylate kinase (AK) and creatine kinase (CK) govern AMP/adenosine (Ado) cycle and the response of metabolic sensors (ATP-sensitive potassium channel, K-ATP and AMP-activated protein kinase, AMPK). Hypoxia or metabolic stress diminishes CK and increases AK flux inducing AMP generation and subsequent AMP/adenosine signaling events. Adenosine/AMP signals delivered to vascular tissue through intercellular and paracellular pathways induce signaling through A(2A) adenosine receptors, AMPK and K-ATP channels. AMPK activates eNOS inducing NO/cGMP signaling and could regulate K-ATP channels. Collectively, A(2A)AR, AMPK, eNOS and K-ATP signaling converge on contractile protein, Ca^2+^ and membrane potential regulation, critical determinants of vascular tone. Dipyridamole, an adenosine uptake inhibitor, disrupts Ado- AMP cycle and tuning of adenosine signals, thus potentiating vascular response. Modified from [4] with permission.

Phosphotransfer reactions have emerged as principle signal generators and coupling relays between cellular metabolism and metabolic sensors [5,29,30,44,218]. In particular, adenylate kinase as generator and modulator of metabolic signals is a critical player integrating complex AMPK and KATP channel signaling cascades in regulation of hormone secretion, blood flow, stress response, muscle and brain functional activity [1,4,5,30,33,34,37,47]. A case in point is myocardial-vascular metabolic signal communication governed by phosphotransfer redistribution, metabolic cycles and relays culminating in a metabolic sensor and functional response (Figure 6) [4]. As brain function specifically depends on glucose metabolism, new spatial and network representation of glycolytic pathway [5] allow for new perspectives and understanding of energetic signal communication and glucose sensing in neurons [30,212].

Body energy sensing depends on information relayed and distributed to a central network of metabolic sensing neurons through hard-wired neural connections and by metabolic and hormonal signals from the periphery coming through the blood and interstitial fluids [212,214]. The brain has a specialized set of neurons that integrate many of these signals and produce regulatory response. These, first described as “glucose sensing” neurons, are really broad-range cellular metabolic sensors that have transporters, metabolite-sensing kinases, ion channels, and receptors that allow them to sense and interpret signals coming from the periphery [214].

Metabolic sensing neurons are integrated into a network that links them to afferent and efferent pathways involved in the control of energy homeostasis [212]. Recent studies indicate that beside satiety hormonal signals, such as leptin and insulin, a myriad of metabolic inputs are generated and sensed by the brain including glucose, fructose, fatty acids and their metabolites, amino acids, Krebs cycle intermediates and nucleotides through specialized receptors and signaling cascades [212–216]. In many cases, reception or metabolism of these signals by sensing neurons results in altered intracellular ATP/AMP ratio and behavior of metabolic sensors such as AMPK, K-ATP channels and other regulators of firing activity [33,72,213,219,220]. This integrated information is then used not only to guide choices the animal makes about the amount of fuels to take in from the environment but also how stored fuels should be distributed and metabolized by various peripheral tissues. Molecular defects or a raise in threshold for sensing catabolic signals from the periphery by specialized metabolic sensing neurons is one of the causes of obesity [212–214].

Metabolism sensing neurons are regulated by glucose through mechanisms requiring either the KATP channel or the Na^+^/glucose cotransporter SGLT3, which is a glucose-sensing rather than glucosetransporting molecule [212–216]. In glucose-excited neurons, the decrease in ATP and ATP-to-ADP ratio leads to activation of K-ATP channels and plasma membrane hyperpolarization, and thus reduction in neuronal firing activity. In glucose-inhibited neurons, the reduced ATP-to-ADP ratio induces closure of chloride channels and/or reduction in the activity of the Na-K pump, depolarization of the plasma membrane, activation of voltage-sensitive Ca^2+^ channels, and synaptic neurotransmitter secretion. Changes in energy expenditure are achieved by regulating hormonal status, sympathetically mediated thermogenesis and cellular energy sensors such as AMPK, which switch off ATP-consuming and activate ATP-regenerating pathways in response to cellular fuel shortage [212–214]. Recent studies show that activity of AMPK is increased in the brain in response to low levels of cellular fuels and negative energy balance and, contrary to peripheral tissues, decreased by leptin [72,213,219]. Furthermore, reduced activity of AMPK in the hypothalamus reduces food intake and body weight [213,219]. Circulating leptin levels give the brain input regarding energy storage so it can regulate appetite and metabolism. Although leptin is a circulating signal that reduces appetite, in general, obese people have an unusually high circulating concentration of leptin and develop leptin resistance [212–214]. Among other factors, consumption of high amounts of fructose causes leptin resistance [221]. This could be due to excess of AMP signaling in the periphery induced by both leptin and fructose which is conveyed to the brain as false “low energy” signal forcing it to increase food consumption. AMP and other peripheral metabolic signals apparently can overcome inhibitory leptin signaling circuits in the hypothalamus [68,153,175]. In this regard, a significant increase in adenylate kinase isoform AK1 and other phosphotransfer enzymes in obese/overweight and morbidly obese women further indicate an imbalance in metabolic signaling in this metabolic syndrome [116].

Labeling studies of adenine nucleotide -phosphoryls indicate that adenylate kinase is active in the brain and that its phosphotransfer rate is increased by drugs improving cerebral circulation and memory dysfunction [222]. Similarly, photic stimulation increases adenylate kinase-mediated ATP β-phosphoryl turnover in photoreceptors suggesting a role in energetics of these specialized cells [223]. Other studies indicate that adenylate kinase isoforms in the brain may contribute to neuronal maturation and regeneration [23,224]. Activation of melanocortin system, which is involved in regulation of appetite, metabolism and body weight, increases expression of adenylate kinase AK1 in the hypothalamus [225]. AMPK, a master metabolic sensor present in hypothalamus, responds to adenylate kinase integrated AMP levels, and plays a critical role in hormonal and nutrient-derived anorexigenic and orexigenic signaling [62,72,226]. Adenylate kinase AK2 is among 14 genes mapped in quantitative trait loci for body weight and abdominal fat weight [227]. Recent proteome studies revealed that adenylate kinase AK2 may be an important regulator involved in the anti-lipid and antioxidant effects of tomato paste [228]. The down-regulation of AK1 expression by hyperglycemia in pancreas may contribute to the defective coupling of glucose metabolism to K-ATP channel activity in type 2 diabetes [48].

Information processing in the brain and energy sensing takes place not only intracellularly but also on extracellular surfaces and between different type of cells through intercellular connections [30,229,230]. In fact, brain ecto-adenylate kinase is an integral part of synaptosomal ATPmetabolizing enzyme cascades regulating ATP, AMP and adenosine signaling [231]. Similarly, adenylate kinase by regulating nucleotide exchange and signaling at peripheral nerve endings can convey information regarding the energy state of particular tissues, thus contributing to body energy sensing. In other cell types ecto-adenylate kinase provides a mechanism for propagation of nucleotide based signals along the cellular surface, thus coordinating multiple receptor-mediated signaling events [64,65]. Extracellular AMP induces bronchoconstriction in suspected asthmatic patients and is used in disease diagnostics as a bronchial provocation test [232]. Both ATP and adenosine signaling which could be modulated by adenylate kinase are critical in synaptic transmission and for normal brain function [233]. Thus, the systemic adenylate kinase → AMP → AMPK signaling network represents a new modality in body energy balance.

In different tissues, adenylate kinase activity depends on the nutritional and hormonal state [234,235]. Adenylate kinase phosphotransfer flux is markedly suppressed by high glucose in insulin secreting cells, reducing adenylate kinase-mediated AMP signaling to the K-ATP channel and AMPK, two regulators of hormone secretion [45,122]. In humans, deficiency of the AK1 isoform is associated with mental retardation, psychomotor impairment and congenital anemia [236]. In this regard, a strong correlation has been observed between fasting and higher expression of the adenylate kinase AK3 isoform, UCP3 and increased activity of AMPK and fatty acid oxidation [237], suggesting an interrelated signaling cascade. Although the significance of energetic and metabolic signaling is growing, little is known regarding the regulation of adenylate kinase phosphotransfer under different metabolic, hormonal and functional states, and how such regulation affects the ability of the cell to generate and respond to energetic stress-related signals.

A new emerging modality in extracellular and intracellular nucleotide signaling and information processing is the cAMP → AMP → adenosine/AMPK pathway sequentially connecting cAMP- and AMP-response elements [238]. In this pathway, cAMP signaling is followed by conversion of cAMP by phosphodiesterases to AMP that activates the AMP-signaling cascade [183]. The role of adenylate kinase in this system is envisioned to propagate AMP metabolic signals along the membrane surface or within the cytosolic space and nuclear compartment where AMPK resides [1,5,65,239]. Cosequently, production of adenosine from AMP by 5′-nucleotidase could stimulate adenosinergic signaling pathways [240]. Thus, this kind of integration of cyclic nucleotide → nucleotide → nucleoside signaling provides means of coordinating diverse signaling events and facilitating information transfer from one subsystem to another.

Understanding principles governing integration and synchronization of metabolic sensors with cellular metabolism is important for regulation of cellular energetic and ionic homeostasis as well as hormonal balance and food intake [37,61,241–243]. Growing evidence and increasing number of discovered metabolic signaling cascades indicate that these systems are essential in vital cellular processes integrating gene expression, metabolism and response to stress, moreover their defects are associated with diseases under a wide umbrella of “metabolic syndrome” [33,62,243,244]. In this regard, recent studies indicate that metabolites linked to glucose such as succinate and alphaketoglutarate can signal through specific G-protein-coupled receptors which are also present in neurons [245,246]. Glucose usually decreases adenylate kinase phosphotransfer flux and intracellular AMP levels and consequently adenosine signaling, but it can increase Krebs cycle substrate levels through anaplerosis [1,246]. Succinate and alpha-ketoglutarate also have signaling function in cell nucleus regulating DNA methylation, which has been implicated in obesity [247]. Taken together, emerging data indicate that coupling of “energetic” phosphotransfer enzymes with phosphoryltransfering protein kinase cascades and metabolite sensitive ion channels, transporters and receptors, comprise a unified intracellular and transcellular energetic and metabolic signal transduction matrix capable of processing, integrating and delivering cellular information regarding energy state (see Figure 6). Within this network, adenylate kinase and AMP signaling throughout the intracellular compartments, extracellular space and body fluids comprise a major metabolic monitoring and body energy sensing node, capable of transducing and distributing signals to metabolic sensors to adjust energy metabolism, fuel selection, food intake and functional response.

## 7. Adenylate Kinase Never Rests: From Altered Energetic Signaling to Immunodeficiency, Cell Motility Defects, Reticular Dysgenesis and Sensorineural Deafness

A thoughtful notion that “adenylate kinase never rests” was expressed by P.B. Detwiler more than a decade ago when commenting on the paper entitled “Adenine nucleoside diphosphates block adaptation of mechano-electrical transduction in hair cells” [248]. In this paper P.G. Gillespie and A.J. Hudspeth demonstrated that mechano-electrical signal transduction and adaptation by hair cells depends on adenine nucleotides and adenylate kinase reaction [248]. Indeed, the past decade further revealed that the dynamic behavior of the adenylate kinase reaction governs many intracellular and extracellular nucleotide signaling processes and that adenylate kinase mutations cause severe human disease phenotypes [1,2,8–11,22,30,102].

Adenylate kinase in conjunction with other phosphotransfer rections is involved in metabolic signaling to membrane ion channels regulating cell ionic balance and electrical activity, and in energy supply to distant ATPases powering spermatozoa and other cell motility related processes [20,29,31,33,44,47]. The large molecular weight isoform AK7 is unusual in the adenylate kinase family; it contains a Dpy-30 motif involved in protein dimerization and is associated with cell motility and other processes [2,5,96]. Mutations in the evolutionarily conserved Ak7 gene results in animals presenting with pathological signs characteristic of primary ciliary dyskinesia (PCD), including ultrastructural ciliary defects and decreased ciliary beat frequency in respiratory epithelium [7]. Ak7 appears to be a marker for cilia with (9 + 2) microtubular organization critical in motility, morphogenesis, cell division and immune response [7,97]. In humans PCD is a genetically and phenotypically heterogeneous disorder, characterized by progressive development of bronchiectasis, inflammation, and features characteristic of chronic obstructive pulmonary disease [7]. Thus, these results suggest that mutations of the human Ak7 gene may underlie a subset of genetically uncharacterized PCD cases.

More recently, the elegant work from B. Wieringa’s laboratory examining the adenylate kinase spatial positioning within a cell by utilizing different artificial location tags, demonstrate that cytoskeleton-based cell motility can be modulated by spatial repositioning of adenylate kinase enzymatic activity to provide local ATP supply and ADP scavenging capacity [8]. These results are corroborated by the use of another heterodimer-inducing approach for transient translocation of AK1 to the specific cellular sites under conditions of constant global AK1 activity in the cell [8]. Thus, adenylate kinase functions as a coupling factor between local ATP supply and regulation of actomyosin and other molecular motor behavior, which is central to cell shape changes and cell motility. Similarly, creatine kinase–mediated ATP supply fuels actin-based events in phagocytosis and is required for thrombin receptor signaling to the cytoskeleton [249,250]. In this regard, the dynamics of actin and myosin in brain presynaptic and postsynaptic regions play a critical role in brain activity and memory formation, and consume the major fraction of the total energy in neurons [251,252]. It remains to be determined whether adenylate kinase provides energy support for motor proteins involved in neuronal trafficking and memory consolidation. Interestingly in humans, genetic adenylate kinase deficiency or losses of adenylate kinase from the brain after surgery are associated with compromise intellectual function [253,254].

Conversion of mechanical stimuli to electrical signals in stereocilia of inner ear hair cells is associated with Ca^2+^ dynamics and movement of myosin motors, similar to many mechanoelectrical and ciliary motility systems [7,248]. The energy supply and nucleotide-based signaling coordinating behavior of such systems is provided by phosphotransfer enzymes including adenylate kinase [1,8,29,30]. It was demonstrated that the creatine kinase circuit is essential for high-sensitivity hearing as demonstrated by hearing loss in creatine kinase knockout mice [255]. Recently biallelic mutations in mitochondrial adenylate kinase AK2 gene have been identified in individuals affected with reticular dysgenesis [9]. Human reticular dysgenesis is the most severe form of inborn severe combined immunodeficiencies (SCID) associated with sensorineural deafness. This disease is characterized by the absence of granulocytes and almost complete deficiency of lymphocytes in peripheral blood. Mutations in AK2 gene result in absent or strong decrease in AK2 protein expression [9]. Restoration of AK2 expression in the bone marrow cells of individuals with reticular dysgenesis overcomes neutrophil differentiation arrest, underlining its specific requirement in the development of a restricted set of hematopoietic lineages. Moreover, AK2 is specifically expressed in the stria vascularis region of the inner ear, which may provide an explanation of the sensorineural deafness in these individuals [9]. Interestingly, immunohistochemistry indicate that AK2 is present within the lumen of the stria vascularis capillaries suggesting that it could be functioning as an ecto-enzyme, however more detailed studies at the cellular level are required and different AK2 and other AK isoform antibodies should be tested. Since almost all cells contain mitochondria and intermembrane AK2, the negative result of intracellular AK2 immunohistochemistry could be related to inaccessibility of intermembrane proteins to antibodies.

It is known that ecto-adenylate kinase plays an important role in extracellular nucleotide signaling; however it is unusual to detect a mitochondrial isoform outside the cell [64,65]. An intriguing possibility exist that beside nucleotide signaling, mucosa embedded AK2 has specific mechanometabolic signal transduction functions and behavior similar to that described in smart hydrogels containing adenylate kinase [256]. A recent proteome study indicates adenylate kinase is present in the extracellular mucus [257]. However, whether the adenylate kinase system due to mutation of AK2 is altered in primary mechanoelectrical signal transducing hair cells which have abundant mitochondria should be determined. Our data indicate that both AK1 and AK2 associate with mitotic spindle, which have microtubular organization (9 + 2) similar to cilia, apparently to power cell division cycle and chromosome disjunction. Previous studies have shown that disruption of analogous adenylate kinase Aky2 gene in yeast coding mitochondrial intermembrane and partially cytosolic AKY2 protein halts ATP export from mitochondria [99]. Thus, AK2 isoform, usually confined within mitochondrial intermembrane space, may have important extramitochondrial functions.

That the gene encoding the mitochondrial energy metabolism related enzyme adenylate kinase 2 (AK2) is mutated in individuals with reticular dysgenesis is supported by simultaneously published independent evidence [10]. Knockdown of zebrafish ak2 gene leads to aberrant leukocyte development, stressing the evolutionarily conserved role of AK2 in leukocyte differentiation [10]. Mononuclear cells obtained from bone marrow of healthy donors lacked AK1, whereas AK2 was readily detectable. These results indicate that leukocytes may be susceptible to defects caused by the lack of AK2, as they do not express AK1 in sufficient amounts to compensate for AK2 functional deficits. Previous studies have linked AK1 mutations to severe nonspherocytic hemolytic anemia and associated with mental retardation and psychomotor impairment [3,236]. In this regard, the deficiency of another metabolic enzyme adenosine deaminase (ADA) accounts for approximately 17% of all SCIDs and 50% of all autosomal recessive SCIDs [258]. The metabolic basis of this immunodeficiency is likely related to accumulation of the ADA substrates adenosine and 2′-deoxyadenosine that kill T and B cells through mechanisms involving accumulation of dATP and induction of apoptosis [258]. Whether nucleotide metabolism is altered in AK2 mutant cells remains to be determined. Taken together, these observations suggest that reticular dysgenesis is the first example of a human immunodeficiency syndrome that is causally linked to the defective phosphotransfer enzyme involved in nucleotide signaling and mitochondrial energy metabolism.

In this regard, absence or reduction of AK2 protein would interfere with mitochondrial bioenergetics and mitochondria-nucleus energetic signal communication that could compromise implementation of leukocyte developmental program [9,30,34]. Evolvement of mitochondrial network and establishment of metabolic circuits are part of developmental programming and execution of cell differentiation sequences [259,260]. A recent study indicates that energy demand signaling gradients arising in the cell would allow propagation of information on local energy consumption over distances, through nucleotide-based signaling conveying positional information to mitochondria [261]. In such way, responding to energy demand gradients, mitochondria can pattern the cytoplasm over length scales that are suited to provide an energy supply continuum and convey morphogenetic information in large cells and tissues [259,261]. Thus, AK2 deficiency can disrupt the flow of developmental information governing cell differentiation.

Adenylate kinase supports energetics and motility of flagella containing parasites and secreted adenylate kinase is a major virulence factor in a number of pathogenic bacteria [5,20,56,57,210]. Recently, a novel myristoylated AK2 isoform has been discovered in P. falciparum causing severe tropical malaria [11]. This modification significantly enhances the stability of the kinase and apparently could be used for targeting the enzyme to membranes or other specific cellular sites, similar to AK1, another myristoylated adenylate kinase isoform [11,51]. The association of myristoylated AK2 with the disease causing clone could be used as a therapeutic target to fight malaria. A large specialized network of six adenylate kinase isoforms exists in a unicellular flagellated parasite Trypanosoma cruzi, the causative agent of Chagas’ disease [59]. This parasite apparently has developed a sophisticated phosphotransfer network where adenylate kinase acts in concert with arginine kinase and nucleoside diphosphate kinase to support the invasive phenotype [262]. Importantly, that mutation in the adenylate kinase gene renders pathogens avirulent [210], suggesting new ways to “silence” otherwise deadly bacteria. Also, a nonstructural protein 4B (NS4B) from hepatitis C virus, which is absolutely required for viral propagation, was found to possess adenylate kinase-like activity [263]. Adenylate kinase 2 is highly upregulated by INF-alpha and IL-15 stimulation in natural killer (NK) cells suggesting role in innate immune defense [264]. In this regard, due to unique catalytic properties and stability adenylate kinase is used as an enzyme amplification system in ATP biosensors for detecting bacterial contaminations in food industry, defense and health care that improves sensitivity levels up to several thousands fold (Celsis, AKuScreen). A recent study demonstrates that mitochondrial AK2 is directly involved in induction of apoptosis through the formation of an AK2-FADD-caspase-10 complex and that downregulation of AK2 attenuates etoposide- or staurosporine-induced apoptosis in human cells [6]. Significantly, that downregulation of cytosolic AK1 transcription with siRNA increases apoptosis in pancreatic cancer cells [265]. Thus, adenylate kinase plays a significant role in making decision between life and death in cellular existence and could be a target in treatment of infectious disease and cancer.

In summary, we highlight here new exciting developments regarding multi-faceted adenylate kinase biology, revealing the significance of mutations and modifications of this never resting phosphotransfer enzyme in energy support of cell motility, disease pathogenesis and regulation of cell differentiation and apoptosis.

## 8. Summary

Metabolic signals regulate and integrate many vital functions throughout the human body, including energy homeostasis, blood pressure, heart performance, food intake, hormonal status and brain performance. Growing evidence indicate the significance of metabolic monitors which directly sense cellular energy state and respond to imbalances by generating and delivering signaling molecules to metabolic sensors to produce a regulatory response. Adenylate kinase-mediated metabolic monitoring and downstream AMP signaling (AK → AMP → AMP-sensors) plays a critical role in the regulation of diverse cellular processes and serves as a primary stress-response pathway. Due to signaling to a number of AMP/nucleoside-sensitive cellular and extracellular components, adenylate kinase senses cellular energetic imbalances caused by physical activity, inadequate oxygenation or nutrient supply, generates and transmits feedback signals to adjust cellular energetics, substrate transport and vascular blood flow to facilitate oxygen and nutrient delivery. Adenylate kinase phosphotransfer dynamics regulates many diverse intracellular and extracellular nucleotide signaling processes, including excitation-contraction coupling, hormonal secretion, cell and ciliary motility, nuclear transport, energetics of cell cycle, DNA synthesis and break repair, and developmental programming. Moreover, adenylate kinase generated and modulated cellular, interstitial and blood AMP levels are emerging as potential metabolic signals that are associated with body energy sensing, sleep, hibernation, vascular flow and food intake. AMP is a mediator of antidiabetic drug action and has growing importance as a therapeutic agent. Within integrated phosphotransfer network, adenylate kinase is essential in integration and synchronization of metabolic sensors with the dynamics of cellular metabolism which is critical for regulation of genetic, energetic, electrical and signal transduction processes determining cell viability and functional activity. As such, adenylate kinase and AMP signaling components dispersed throughout the intracellular compartments, extracellular spaces and body fluids comprise a major metabolic monitoring and body energy sensing node transducing and distributing signals to metabolic sensors, thus conveying information about body energy and fuel usage status. Moreover, evidence is mounting regarding the direct relationship between defects in adenylate kinase and AMP metabolic signaling and human diseases, such as heart failure, hypertrophic cardiomyopathy, diabetes, obesity, hemolytic anemia, reticular dysgenesis, ciliary dyskinesia, cancer and neurodegeneration. Thus, adenylate kinase, previously considered as a regular housekeeping enzyme, is a critical player in metabolic monitoring and systemic integration of different signaling pathways to ensure cellular energy homeostasis and an adequate response to a broad range of functional, environmental and stress challenges.
